# The Wywodzof Method of Preserving Subjects

**Published:** 1881-04

**Authors:** 


THE WYWODZOF METHOD OF PRESERREING SUBJECTS.—This method was used to embalm the body of Anson Burlingame and succeeded so well that, several years later the body was found in an excellent stata of preservation. He makes few incisions and does not open the cavity of the abdomen or thorax. He opens only the carotid and crural arteries, and after many experiments has reached the following conclusions (*Med. and Surg. Reporter:*) The best lixuid to inject into a subject, a limb or a vesical organ, is the following solution: Glycerine, 4 lbs.; water, 2 lbs.; thymol, 3 grams. (2) A quantity of liquid about one-half the weight of the subject is necessary. (3) The liquid should be slowly injected. If one limb is to be injected, the denuded part should be immersed in boiling water and the medullary canal stopped up with a cork. (4) The veins accompanying the carotid and femoral arteries, the jugular and femoral, should be opened near the openings in the arteries and the liquid injected until it appears in these veins. (5) When the liquid commences to pass by mouth of the subject the trachea must be opened, a cork inserted and a stout ligature applied. (6) When the capillaries of the skin are filled the operation may be considered finished. If one of the limbs should appear not well done, it should be separately injected.—*Detroit Lancet.*

